# The conserved ancient role of chordate PIAS as a multilevel repressor of the NF-κB pathway

**DOI:** 10.1038/s41598-017-16624-7

**Published:** 2017-12-06

**Authors:** Ruihua Wang, Shengfeng Huang, Xianan Fu, Guangrui Huang, Xinyu Yan, Zirui Yue, Shangwu Chen, Yingqiu Li, Anlong Xu

**Affiliations:** 1 State Key Laboratory of Biocontrol, Guangdong Key Laboratory of Pharmaceutical Functional Genes, School of Life Sciences, Sun Yat-sen University, Guangzhou, 510275 People’s Republic of China; 2 Center for Regenerative and Translational Medicine, Guangdong Provincial Academy of Chinese Medical Sciences, the Second Affiliated Hospital of Guangzhou University of Chinese Medicine, Guangzhou, 510632 People’s Republic of China; 3 School of Life Sciences, Beijing University of Chinese Medicine, Dong San Huang Road, Chao-yang District, Beijing, 100029 People’s Republic of China

## Abstract

In vertebrates, *PIAS* genes encode versatile cellular regulators, with functions extremely complex and redundant. Here we try to understand their functions from an evolutionary perspective. we evaluate the sequences, expression and molecular functions of amphioxus *PIAS* genes and compare them with their vertebrate counterparts. Phylogenetic analysis suggests a single *PIAS* gene in ancestral chordates, which has been duplicated into four families (*PIAS1-4*) in vertebrates by 2R-WGD but remained single in a basal chordate (amphioxus). Amphioxus *PIAS* encodes two variants with and without a Serine/Threonine-rich tail, which are retained in human *PIAS1-3* but lost in *PIAS4*. We show that amphioxus PIAS binds C-terminus of NF-κB Rel and blocks the DNA binding activity. In humans, such function is retained in PIAS1, altered in PIAS4, and lost in PIAS2-3. Instead, PIAS3 has evolved new ability to inhibit Rel by binding RHD and promoting SUMOylation. We show that amphioxus PIAS also inhibits NF-κB by binding with upstream signalling adaptor TICAM-like and MyD88. Finally, we verify that human PIAS1, 3 and 4, but not 2, were capable of these newly-discovered functions. Our study offers insight into the sub- and neo-functionalization of *PIAS* genes and suggests a conserved ancient role for chordate PIAS in NF-κB signalling.

## Introduction

Protein inhibitors of activated STATs (PIAS) are versatile cellular regulators^1–3^. There are four *PIAS* gene families in vertebrates, including *PIAS1*, *PIAS2* (*PIASx*), *PIAS3* and *PIAS4* (*PIASy*)^4–8^. So far, these families have been proposed to regulate the function of over 60 proteins^1^, and these regulations are conducted through various molecular mechanisms^9^. For example, PIAS proteins affect transcription by blocking the DNA binding activity of transcription factors (e.g., STAT1), by recruiting co-regulators (e.g., ZNF133), or by altering the subcellular localization of target proteins (e.g., SNM1A)^5,10,11^. PIAS proteins are key small ubiquitin-related modifier protein (SUMO) E3 ligases that modulate the function and subcellular localization of many proteins (SMAD4, p73, MDM2, etc.) by promoting SUMOylation^1^. PIAS proteins are notorious for functional redundancy. For example, both PIAS1 and PIAS4 inhibit STAT1 and PLAG1^5,12,13^; both PIAS1 and PIAS2 regulate SMAD4, p53, p73, JUN, MDM2, AR and C/EBPε^1^; and PIAS2, PIAS3 and PIAS4 suppress C/EBPδ^14^. Moreover, possibly due to functional compensation from other *PIAS* genes, *PIAS4* ^−/−^ mice display no serious defects^15,16^. The study of vertebrate *PIAS* genes has been hindered by functional complexity and redundancy. To gain a better understanding of the PIAS functions, here we try to analyse the functional origin and evolution of vertebrate *PIAS* genes.

Nuclear factor-κB (NF-κB) transcription factors participate in many biological processes, but it has been proposed that their most central and evolutionarily conserved role is to regulate the development and functions of the immune system^17,18^. There are a number of receptors (TNFRs, IL-1Rs, TLRs, NLRs, RLRs, etc.) and signal transducers (TAB2, RIP1, IRAKs, TRAFs, MyD88, TRAM, TRIF, IKKs, etc.) participating in NF-κB activation^19^. In addition, NF-κB signalling is also regulated by post-translational modification mechanisms, such as ubiquitination and SUMOylation^20–22^. There is emerging evidence showing that PIAS proteins can serve as regulators of the NF-κB pathway in vertebrates. For example, PIAS1, PIAS3 and PIAS4 interact with NF-κB RelA to inhibit its transcriptional activity; PIAS3 represses NF-κB by promoting TAB2 SUMOylation; and PIAS4 blocks TRIF from activating NF-κB^23–28^. In response to genotoxic stress, PIAS4 can facilitate NF-κB activation by targeting NF-kappaB essential modulator (NEMO) to the nucleus by promoting NEMO SUMOylation^29^. To date, we still do not fully understand the roles of PIAS proteins in NF-κB signalling.

Amphioxus (*Branchiostoma*) represents the most basally divergent living chordate lineage that diverged from the other two chordate lineages (vertebrates and urochordates) more than half a billion years ago^30^. Amphioxus retained many chordate ancestral features and did not undergo the two rounds of whole genome duplication (2R-WGD) that vertebrates underwent early in their evolution^31,32^. Therefore, amphioxus is considered one of the best available proxies for ancestral vertebrates^33–35^. Amphioxus has a simplified NF-κB pathway compared with vertebrates^36,37^. Transcriptomic and functional analyses have confirmed that NF-κB plays a critical role in regulating immune responses in amphioxus^37,38^. So far, dozens of receptors and signal transducers related to NF-κB activation have been examined in amphioxus (reviewed in^39^). However, how PIAS regulates NF-κB signalling in amphioxus remains elusive. Here, our study on amphioxus *PIAS* not only reveals some new functions of *PIAS* genes but also offers insight into the functional consequences regarding the evolution of vertebrate *PIAS* genes.

## Results

### One *PIAS* gene in amphioxus versus four in vertebrates

**Figure 1:**
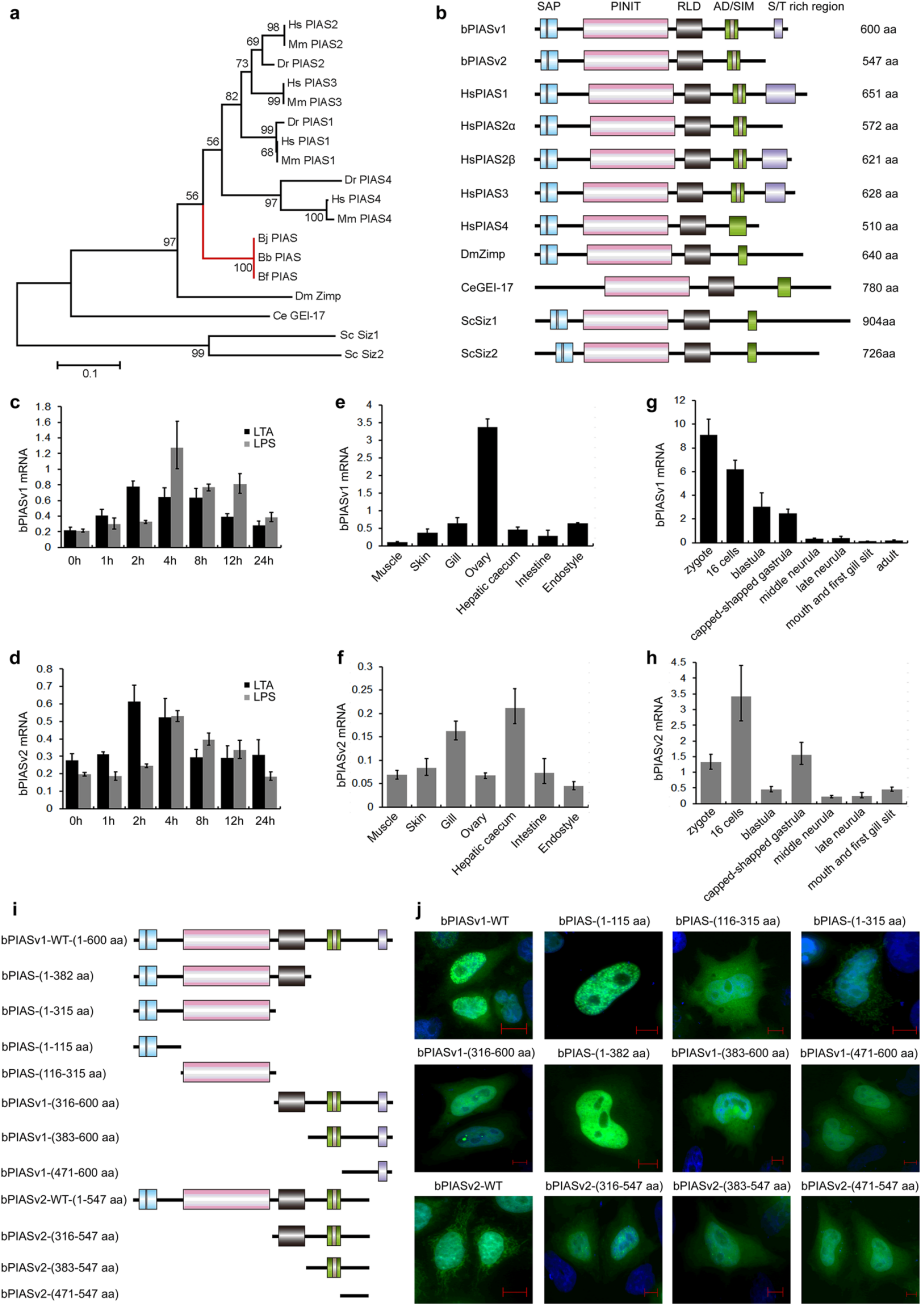
The sequence, expression and cellular localization of amphioxus PIAS. (**a**) Phylogenetic analysis of PIAS proteins based on the highly conserved RLD domain. The tree was inferred using MEGA7 with the Maximum Likelihood method and 500 bootstrap tests. (**b**) The protein architecture of PIAS family members. The sequence length is indicated. (**c**,**d**) qRT-PCR analysis of the time course of *bPIASv1* and *v2* mRNA expression dynamics after challenge with LTA and LPS. Note that the 0 h of treatment is equivalent to the unchallenged animals. (**e**,**f**) qRT-PCR analysis of *bPIASv1* and *v2* mRNA expression in different tissues. (**g**,**h**) qRT-PCR analysis of dynamic *bPIASv1* and *v2* mRNA expression during amphioxus embryogenesis. The data were expressed as a ratio between *bPIASv1* or *v2* mRNA and the mRNA expression level of GAPDH. (**i**) The truncated mutants of bPIAS used in this study. (**j**) The subcellular localization of full-length bPIASv1 and v2 and the associated truncated mutants. The scale bar indicates 10 µm.

We examined the draft genomes and transcriptomes of three amphioxus species (*Branchiostoma japonicum*, *B. belcheri* and *B. floridae*) and confirmed that there is only one single *PIAS* gene orthologue (designated *bPIAS*, hereafter). In contrast, vertebrates contain four *PIAS* gene families. The tree topology revealed by phylogenetic analysis with the most conserved domain (RLD) suggests that the *PIAS* gene was duplicated into four copies early in vertebrate evolution through 2R-WGD (Fig. 1a). Synteny analysis confirms this conclusion. We should point out that if the full-length sequence of PIAS genes were used for phylogenetic analysis, the tree topology would be changed due to the accelerated evolutionary rates in the non-conserved regions of *PIAS4* (see Supplementary Fig. S1). We know that most of the paralogs derived from 2R-WGD have been lost in modern vertebrates. Keeping all four paralogs may have caused complicated interactions and redundancy, as is exactly the case in *PIAS* paralogs (as we discussed in the Introduction part). In this sense, amphioxus has a much more simplified PIAS interaction network than do vertebrates.

### Amphioxus *PIAS* encodes both S/T-rich and non-S/T-rich C-terminals

A typical domain architecture for PIAS proteins includes an N-terminal scaffold attachment factor A/B, acinus and PIAS domain (SAP domain), a Por-Ile-Asn-Ile-Thr (PINIT) domain, an RLD (RING finger–like zinc-binding domain) and an AD (highly acidic domain). In addition, vertebrate PIAS1, 2 and 3 have a C-terminal S/T-rich region, but this region is absent in vertebrate PIAS4 and in the PIAS proteins of *Drosophila melanogaster* and *Caenorhabditis elegans* (Fig. 1b). Therefore, having no S/T-rich region could be the ancestral state for PIAS proteins, whereas possessing the S/T-rich region could be a new vertebrate feature. Here, we show that despite being a single-copy gene, amphioxus *PIAS* produces two protein variants through alternative splicing. Both bPIASv1 (600 aa) and bPIASv2 (547 aa) have a typical PIAS protein domain architecture, but they have different C-terminal sequences after amino acid 478, with bPIASv1 containing an S/T-rich tail and bPIASv2 having none (Fig. 1b). These findings suggest that the origin of the S/T-rich tail could be traced back to the chordate ancestor. In amphioxus, PIAS switch between S/T-rich and non-S/T-rich tails by alternative splicing, while in vertebrates, PIAS1 and 3 have the S/T-rich tail, PIAS4 has the non-S/T-rich tail, and only PIAS2 retain both tails by alternative splicing.

### Different roles for amphioxus PIASv1 and PIASv2

Quantitative RT-PCR was conducted to measure the *in vivo* mRNA expression of *bPIASv1* and *v2*. In response to immune stimulations with lipoteichoic acids (LTA) and lipopolysaccharides (LPS), both bPIAS variants were up-regulated in the amphioxus gut, suggesting their role as negative feedback regulators for gut immunity (Fig. 1c,d).

Regarding tissue distribution, *bPIASv1* but not *v2* showed predominant abundance in ovaries (Fig. 1e,f). Similarly, mouse *PIAS2* is abundant in testes, and *PIAS2* ^−/−^ mice show reduced testes and sperm counts^40,41^. This suggests that a role in gamete production is conserved between *bPIAS* and vertebrate *PIAS2*.

During amphioxus embryogenesis, *bPIASv1* presented as maternal transcripts in abundance and gradually reduced as time lapsed, while *bPIASv2* was up-regulated in the 16-cell and gastrula stages (Fig. 1g,h). This suggests distinct roles of the two amphioxus PIAS variants in embryogenesis. Similarly, PIAS is essential for embryogenesis in both *D. melanogaster* and *C. elegans* ^42,43^. This suggests an ancient role in development for both chordate and non-chordate PIAS proteins. Indeed, vertebrate PIAS proteins are apparently involved in embryogenesis, but their roles are somewhat concealed by functional redundancy between PIAS1, 2, 3 and 4^7,15,27^. As a single-copy gene, *bPIAS* may have advantages in revealing the developmental role of vertebrate *PIAS* genes.

### Nuclear retention of amphioxus PIAS is determined by SAP but not PINIT

To determine the subcellular localization of bPIAS, full-length or truncated bPIAS was fused with GFP (Fig. 1i) and transfected into HeLa cells. As shown in Fig. 1j, overexpressed full-length bPIASv1 and bPIASv2 were localized in the cytoplasm as filamentous structures and in the nucleus as small punctate structures. In comparison, overexpressed mammalian PIAS4 localized to the nucleus as punctate structures, whereas endogenous mammalian PIAS1 and PIAS4 were uniformly distributed in the nucleus and in the cytoplasm at a lower concentration^12,24,44^. Further analysis with truncated mutants demonstrated that the PINIT domain, considered essential for the nuclear retention of mouse PIAS3^45^, plays no role in the nuclear localization of bPIAS. Instead, the N-terminal 1–115 aa that contain the SAP domain (capable of binding nuclear matrix chromatins) are responsible for retaining bPIAS in nuclei (Fig. 1j).

### Amphioxus PIAS inhibits the NF-κB activation induced by various signals

**Figure 2:**
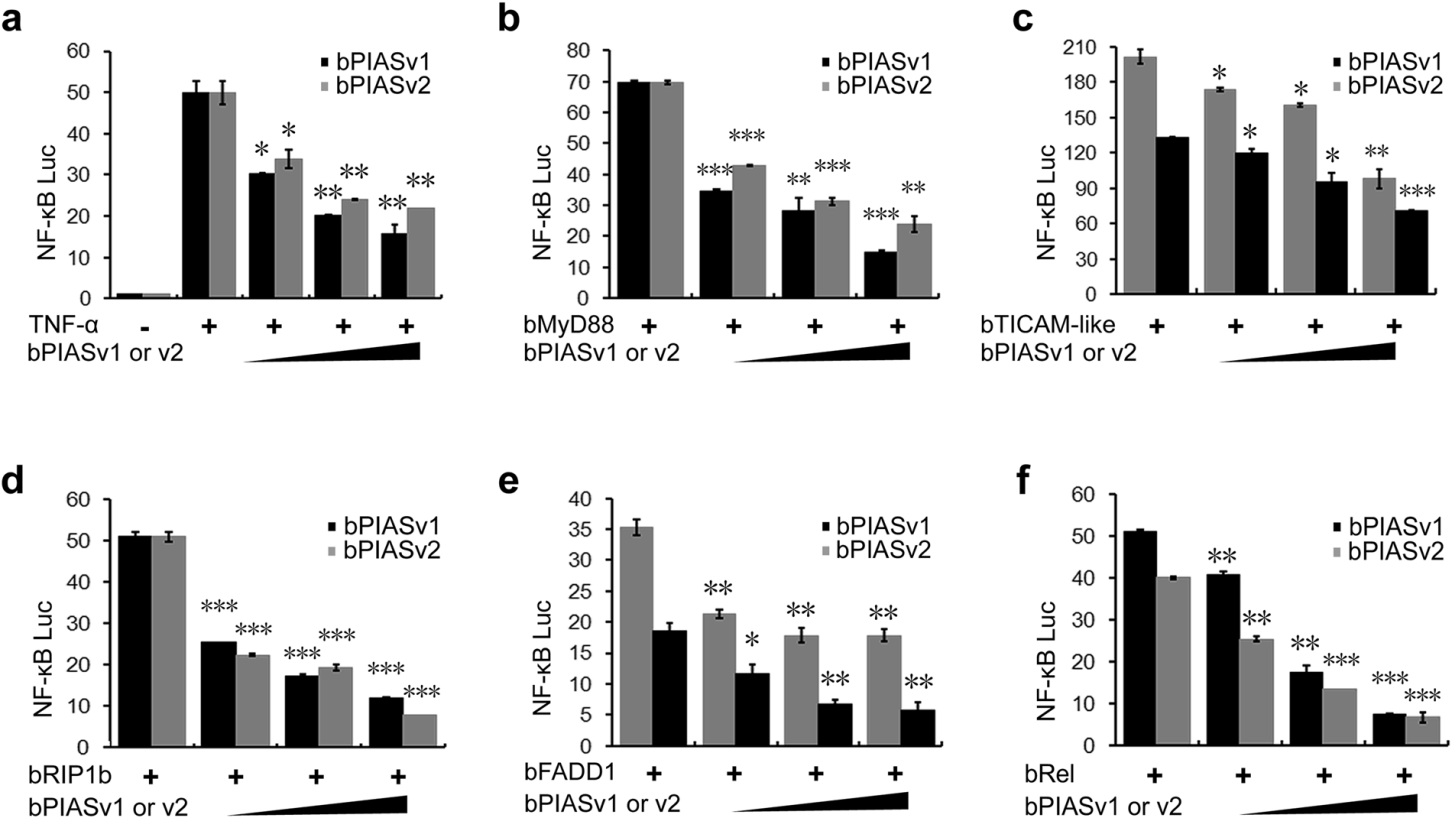
Amphioxus PIAS inhibits NF-κB activation induced by different signals. (**a**–**f**) Luciferase reporter assays show that bPIAS can repress NF-κB activation mediated by the cytokines TNFα and several NF-κB related signal transducers of amphioxus in HEK293T cells. All reporter assays were performed in triplicate and repeated at least twice. Values are expressed as the mean ± SD; *P < 0.05, **P < 0.01, ***P < 0.001.

Mammalian PIAS1, 3 and 4 repress NF-κB transactivation induced by surface immune receptors (TNFRs, IL-1Rs and TLRs) and cognate signal transducers^23–28^. To investigate whether amphioxus PIAS could inhibit NF-κB activation, human 293 T cells were transfected with NF-κB-luciferase reporter plasmids together with empty plasmids or bPIAS expression plasmids. At 24 hours post-transfection, cells were incubated with or without human TNFα for 7 hours, and then NF-κB activity was measured. As shown in Fig. 2a, both bPIASv1 and v2 inhibited TNFα-mediated NF-κB activation in a dose-dependent manner. Further reporter assays showed that when expressed in human cells, several NF-κB-related signal transducers of amphioxus, including bMyD88, bTICAM-like (a homologue to human TRAM/TRIF), bRIP1, bFADD1 and bRel, could significantly activate NF-κB dependent transcription. However, all of these signal transducers could be suppressed by both bPIASv1 and v2 in a dose-dependent fashion (Fig. 2b–f). These results show that the amphioxus NF-κB regulatory machinery is functional in human cells and that the amphioxus PIAS, similar to its vertebrate counterparts, is a repressor of NF-κB signalling.

### Amphioxus PIAS interacts with NF-κB Rel but not IκB

**Figure 3:**
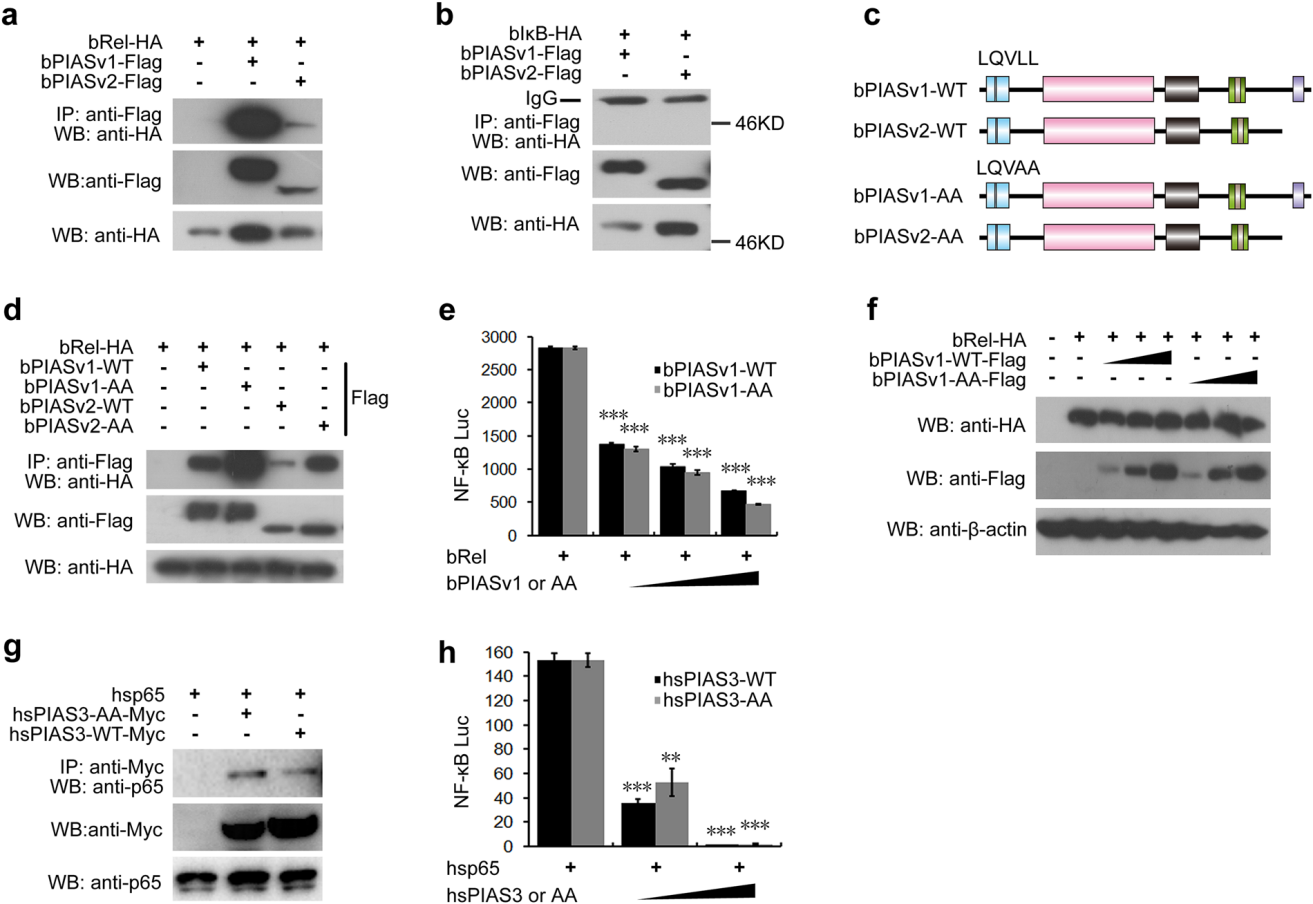
Amphioxus PIAS interacts with NF-κB Rel, and the LXXLL motif in bPIAS is not required for binding and inhibiting bRel. (**a**,**b**) Co-IP analyses of the interactions between bPIAS and bRel (**a**) or bIκB (**b**). (**c**) The bPIAS-(LL-AA) point mutants used in this study. (**d**) Co-IP assays indicate that the bPIAS-AA point mutant is also capable of binding to bRel, similar to its wild-type counterpart. (**e**) Luciferase reporter assays show that bPIAS-AA can inhibit bRel-dependent transcription activation to the same extent as the wild-type bPIAS. (**f**) Western blot was performed to show the expression of relevant proteins, including bPIAS-WT, bPIAS-AA and bRel, β-actin was used as the internal reference. (**g**) Co-IP assays indicate that the hsPIAS3-AA point mutant is also capable of binding to hsp65, similar to its wild-type counterpart. (**h**) Luciferase reporter assays show that hsPIAS3-AA can inhibit hsp65-dependent transcription activation to the same extent as the wild-type hsPIAS3. Data show a representative result from at least three separate experiments. ^*^P < 0.05, ^**^P < 0.01, ^***^P < 0.001.

Described above, bPIAS was shown to inhibit various NF-κB activation signals, which could be achieved by acting on IκB/NF-κB proteins because diverse activation signals eventually converge on IκB/NF-κB proteins. Previous studies demonstrated that amphioxus have one orthologue (bIκB) for human IκB proteins and one orthologue (bRel) for human short-form NF-κB proteins RelA, RelB and C-Rel^37,38^. To test whether bPIAS could directly interact with bRel and bIκB, we conducted coimmunoprecipitation (Co-IP) assays. Flag-tagged bPIAS was co-transfected with HA-tagged bRel into 293 T cells; then, cell lysates were immunoprecipitated with anti-Flag mAb, and the coprecipitated proteins were examined with anti-HA mAb. As shown in Fig. 3a, both bbtPIASv1 and v2 could bind to bRel. In similar assays, bPIAS failed to associate with bIκB (Fig. 3b). These results show that bPIAS interacts with bRel but not bIκB. This is consistent with earlier reports in which mammalian PIAS1, 3 and 4 have been shown to negatively regulate NF-κB activation by directly interacting with RelA^24,25,27^.

### The LXXLL motif in bPIAS is not required for binding and inhibiting bRel

The α-helical LXXLL signature motif of the SAP domain can mediate interactions between nuclear receptors and their co-regulators^46^, which is essential for PIAS4 to repress STAT1 and androgen receptors^12,47^ and for PIAS3 to bind the RHD of RelA and inhibit RelA activation^25^. We identified a conserved LQVLL motif in the SAP domain of bPIAS. To investigate the function of this motif, we mutated it to LQVAA as a previous study did to mouse PIAS3 (Fig. 3c). Subsequent Co-IP and luciferase assays showed that unlike mouse PIAS3^25^, the mutation did not compromise the ability of bPIAS to bind and inhibit bRel (Fig. 3d–f), this is also consistent with the result of human PIAS3(Fig. 3g,h), suggesting that, unlike mouse PIAS3, the LXXLL motif of both bPIAS and human PIAS3 is not required for binding and inhibiting Rel.

Taken together, our current results show that bPIAS and mammalian PIAS1 use a similar mechanism to repress the functions of NF-κB Rel. This mechanism could be considered as a conserved ancient mechanism for chordate PIAS proteins. On the other hand, mammalian PIAS3 has developed a new mechanism to inhibit RelA.

### Amphioxus PIAS blocks the DNA binding ability of bRel

**Figure 4:**
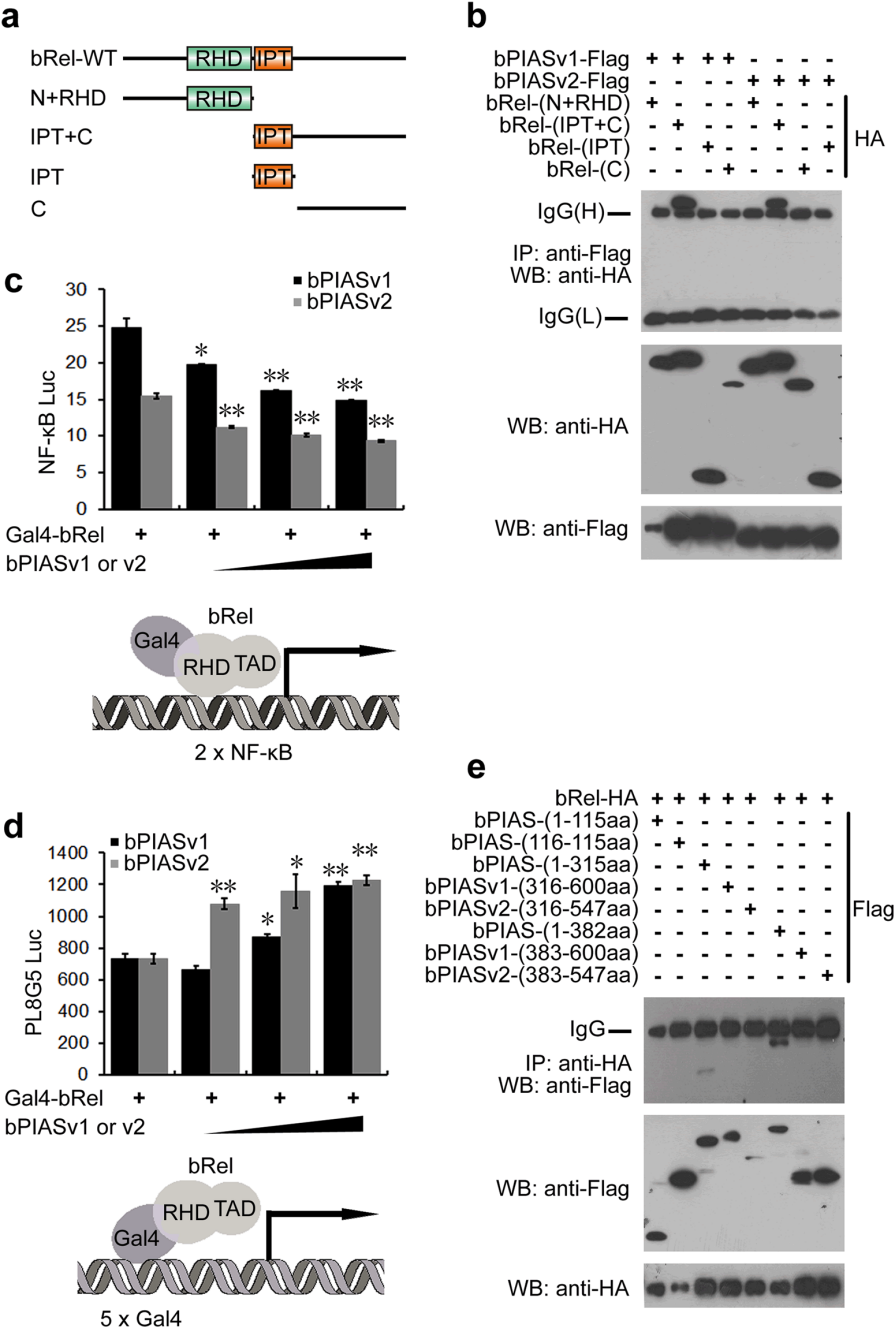
Amphioxus PIAS blocks the DNA binding ability of NF-κB Rel. (**a**) Domain structures and various truncated variants of bRel used in this study. (**b**) Co-IP analyses of the interactions between bPIAS and the truncated mutants of bRel. (**c**,**d**) Luciferase reporter assays were performed in HEK293T cells with an expression construct encoding Gal4-bRel fusion protein, increasing amounts of bPIAS, and either 2 × NF-κB reporter (**c**) or 5 × Gal4 reporter (**d**). (**e**) Co-IP analyses of the interactions between the truncated mutants of bPIAS and bRel. All experiments were conducted at least twice. All reporter assays were performed in triplicate and repeated at least twice. Values are expressed as the mean ± SD; ^*^P < 0.05, ^**^P < 0.01, ^***^P < 0.001.

Rel proteins contain an N-terminal Rel homology domain (RHD), an immunoglobulin-like fold, Plexins, Transcription factors (IPT) domain and a C-terminal transactivation domain (TAD). Mammalian PIAS1 blocks the DNA binding of RelA by interacting with the TAD, while mammalian PIAS3 inhibits RelA by binding to the RHD and promoting SUMOylation^24,25^. To determine which region of bRel could interact with bPIAS, several truncated forms of bRel were constructed and analysed using Co-IP assays in 293 T cells. As shown in Fig. 4a,b, the C-terminal region (IPT + TAD) of bRel was responsible for its binding with both bPIASv1 and v2. Then, we constructed a Gal4-bRel fusion protein, which could activate luciferase reporters with either a 5 × Gal4 binding site or a 2 × NF-κB binding site. When bPIAS and Gal4-bRel expression plasmids were co-transfected into 293 T cells with 2 × NF-κB reporter plasmids, both bPIASv1 and v2 were shown to inhibit the bRel-dependent transactivation in a dose-dependent fashion (Fig. 4c), which suggests that bPIAS blocks the DNA binding activity of bRel. However, when bPIAS and Gal4-bRel expression plasmids were co-transfected with 5 × Gal4 reporter plasmids, they were shown to not inhibit but activate the bRel-dependent transactivation of the 5 × Gal4 reporter (Fig. 4d). One explanation of this result is that, because bPIAS predominantly exists in the nucleus, when co-transfected with Gal4-bRel, bPIAS could interact with bRel. Therefore, increasing the amount of bPIAS may draw more Gal4-bRel into the nucleus through PIAS-Rel interactions, and Gal4-bRel, in return, bind to the Gal4-binding site and activate the luciferase reporter. Additional Co-IP assays demonstrated that the N terminus of bPIAS (aa 1–315) containing the SAP and PINIT domains was the minimal requirement for binding with bRel (Fig. 4e). These results demonstrate that bPIAS blocks the DNA binding activity of bRel through interactions between the SAP + PINIT of bPIAS and the IPT + TAD of bRel.

### Both amphioxus and human PIAS bind to MyD88 and TICAM-like

We performed analyses to determine whether bPIAS could interact with other upstream signal transducers of the NF-κB pathway. Our results suggest that both bMyD88 and bTICAM-like can directly associate with bPIAS.

**Figure 5:**
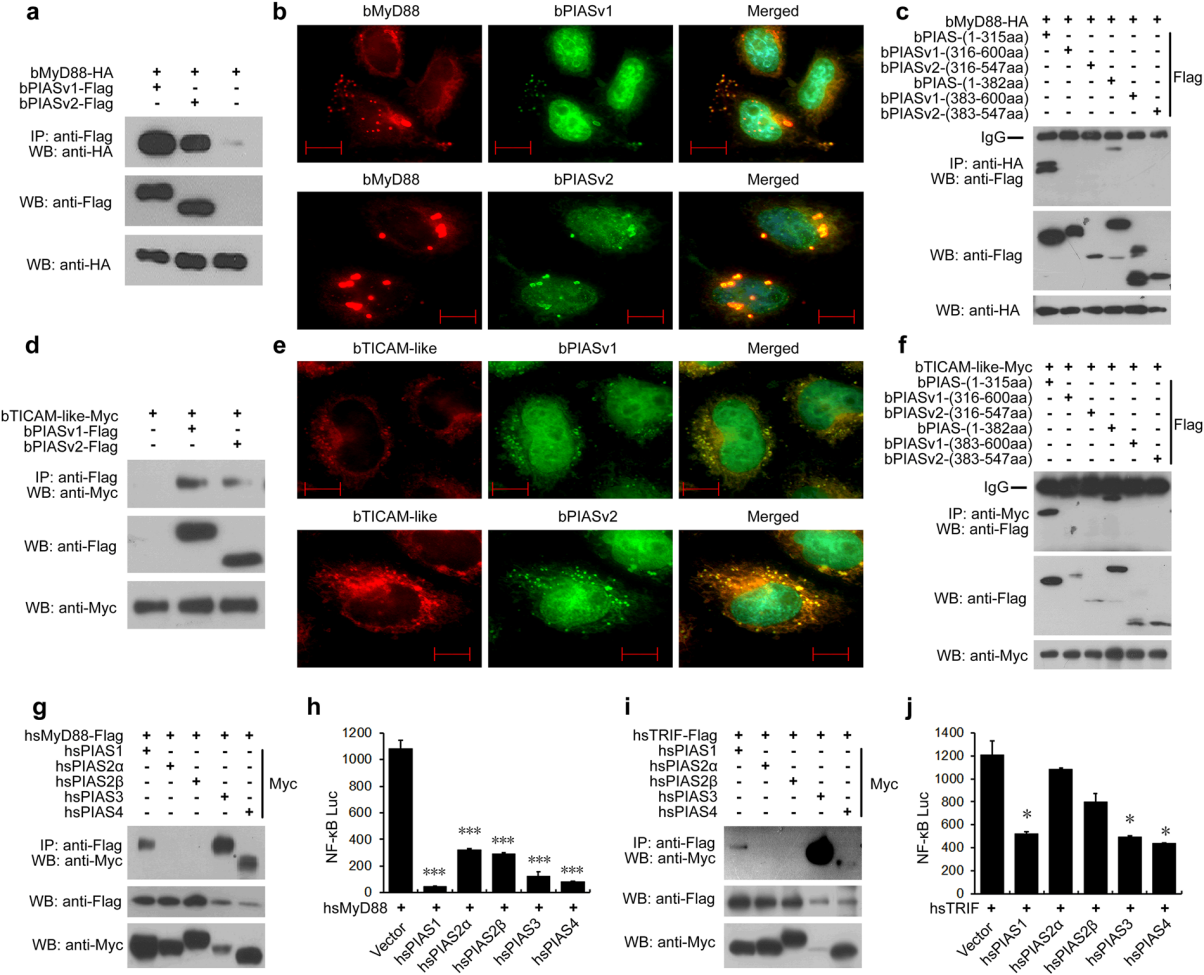
Both amphioxus and human PIAS can bind to MyD88 and TICAM-like and suppress NF-κB activation. (**a**) Co-IP analyses of the interactions between bPIAS and bMyD88. (**b**) Immunofluorescence analysis of the subcellular co-localization of amphioxus PIAS and MyD88 in HeLa cells, which were co-transfected with EGFP-fused bPIASv1 or v2 with HA-tagged bMyD88 and stained with anti-HA antibody and an Alexa Fluor 532 secondary antibody. Scale bar indicates 10 μm. (**c**) Co-IP analyses of the interactions between the truncated mutants of bPIAS and bMyD88. (**d**) Co-IP analyses of the interactions between bPIAS and bTICAM-like. (**e**) Immunofluorescence analysis of the subcellular co-localization of amphioxus PIAS and bTICAM-like in HeLa cells, which were co-transfected with EGFP-fused bPIASv1 or v2 with Myc-tagged bTICAM-like and stained with anti-Myc antibody and an Alexa Fluor 532 secondary antibody. The scale bar indicates 10 µm. (**f**) Co-IP analyses of the interactions between the truncated mutants of bPIAS and bTICAM-like. (**g**) Co-IP analyses of the interactions between human PIAS and MyD88. (**h**) Luciferase reporter assays indicate that human PIAS1, 3 and 4 can attenuate hsMyD88-induced NF-κB activation more robustly. (**i**) Co-IP analyses of the interactions between human PIAS and TRIF. (**j**) Luciferase reporter assays show that human PIAS1, 3 and 4 but not PIAS2 can repress NF-κB activation mediated by hsTRIF. All experiments were conducted at least twice. Data in (**h**) and (**j**) are means ± SD for 3 independent experiments. ^*^P < 0.05, ^***^P < 0.001.

Amphioxus MyD88 is the only orthologue of human MyD88. Co-IP assays in 293 T cells indicated interaction between bMyD88 and both bPIAS variants (Fig. 5a). Immunofluorescence analysis in HeLa cells confirmed that bPIAS and bMyD88 co-localized in the same cytoplasmic punctate structures (Fig. 5b). Further Co-IP assays suggested that 1–315 aa of bPIAS was the minimal requirement for binding with bMyD88 (Fig. 5c).

Amphioxus TICAM-like is a distant homologue to human TRIF (also named TICAM-1) and TRAM (also named TICAM-2). Co-IP assays in 293 T cells showed that bTICAM-like interacted with both bPIAS variants (Fig. 5d). Immunofluorescence analysis confirmed the co-localization of bPIAS and bTICAM-like in HeLa cells as small punctate structures (Fig. 5e). Further Co-IP assays suggested that 1–315 aa of bPIAS was the minimal requirement for binding with bTICAM-like (Fig. 5f).

Since there are no previous reports on the interaction between PIAS and MyD88, we carried out assays to determine whether this interaction is present in human PIAS proteins. The results showed that human PIAS1, 3 and 4 but not PIAS2 could interact with human MyD88 and inhibit the activation of NF-κB (Fig. 5g,h). On the other hand, there have been studies demonstrating that vertebrate PIAS4 can bind to TRIF and repress TRIF-induced NF-κB activation^6,26^. However, it was not known whether other human PIAS proteins can also interact with TRIF. We carried out assays to determine the interactions between human PIAS proteins and TRIF. The results showed that in addition to PIAS4, human PIAS1 and 3, but not PIAS2, could also interact with human TRIF and inhibit the activation of NF-κB (Fig. 5I,j). Taken together, our data suggest that PIAS-MyD88 and PIAS-TRIF interactions are conserved throughout chordate evolution.

## Discussion and Conclusions

In this study, we show that the *PIAS* gene remained as a single-copy in the basal chordate amphioxus but was duplicated into four paralogs (*PIAS1-4*) in vertebrates through 2R-WGD. Unlike most paralogues that have been lost in later evolution, all four *PIAS* paralogues are preserved in human and other vertebrates. These *PIAS* genes still share functional redundancies but have also developed complicated new functions, thereby making it difficult to unravel their biology.

There is a general model to explain the functional consequences of the evolution of duplicated genes^48,49^. In this model, after gene duplication, a paralog loses a part of its function (nonfunctionalization) due to functional redundancy so that, in the long run, different paralogs will retain different parts (subfunctionization) of the ancestral function. This process relaxes selection constraints and therefore allows paralogs to gain new functions (neofunctionalization).

**Figure 6:**
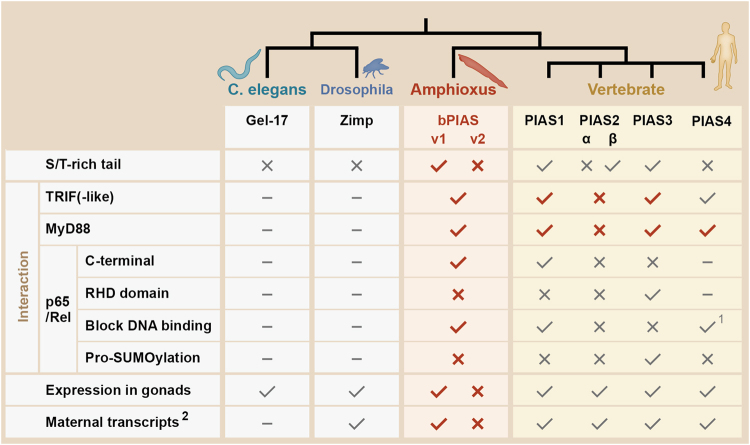
Known functional consequences of the evolution of chordate PIAS genes. The symbols √, × and - indicate the status of “yes”, “no” and “not available/not tested”, respectively. The contents highlighted in red indicate new discoveries from this study. ^1^PIAS4 binds with NF-κB Rel and cooperates with PIAS1 to regulate the specificity and magnitude of NF-κB-mediated gene activation^27^. ^2^The evidence that all four vertebrate PIAS genes are expressed maternally during early embryogenesis came from *Xenopus laevis* ^7^.

Our comparative analysis suggests that the repression of NF-κB signalling at multiple levels by both amphioxus and human PIAS proteins is a conserved role in chordates. In amphioxus, we show that the single PIAS orthologue can negatively regulate NF-κB activation through binding with TICAM-like and MyD88, and blocking the DNA binding activity of NF-κB Rel (Fig. 4). In humans, we observe substantial subfunctionalization and neofunctionalization in *PIAS1-4* (Fig. 6). For example, amphioxus *PIAS* encodes both S/T-rich and non-S/T-rich C termini through alternative splicing. In vertebrates, *PIAS1-3* only encodes the S/T-rich tail and that PIAS4 only encodes the non-S/T-rich tail. We reveal that human PIAS1, 3 and 4 also have the ability to interact with TICAM-1 and MyD88, while PIAS2 has lost this ability. Human PIAS1 and amphioxus PIAS use the same mechanism to block the DNA binding activity of RelA/Rel, but there is no evidence that human PIAS2 is capable of such an interaction. Remarkably, unlike human PIAS1 and 4, which tend to interact with the C terminus of RelA^24,27^, human PIAS3 interacts with the N terminus of RelA and inhibits RelA by promoting its SUMOylation^23,25^, which could be considered neofunctionalization after nonfunctionalization.

Comparative analysis using amphioxus has shed new light on the implications of vertebrate *PIAS* genes in NF-κB signalling, but it also raises new questions. One important question is how to understand the biological meaning of the pleiotropy of PIAS. Here, we confirm that human PIAS proteins can interact with TRIF/TICAM-1, MyD88 and RelA. In addition, mouse TAB2, another upstream signal transducer of NF-κB signalling, has also been found to be inhibited by PIAS3-mediated SUMOylation^28^. Therefore, the reason why PIAS proteins regulate the NF-κB pathway at different levels is fascinating. There are some preliminary ideas and information. Unlike IκB or other NF-κB repressors, PIAS proteins only regulate a subset of NF-κB-mediated proteins^24,27^. Different terminal pathways, such as STATs, NF-κBs, AP-1, IRFs and caspases, share overlapping upstream adaptors and have crosstalk. The ability to interact with both upstream adaptors and transcription factors may allow PIAS proteins to coordinate the crosstalk and to properly fine tune signalling under various physiological conditions^3,9^. Indeed, PIAS proteins can be highly flexible and discriminative in certain situations. For example, in many situations, PIAS4 acts as a repressor of NF-κB activation induced by TNFα, LPS and IL-1β^27^, but in response to genotoxic stress, it facilitates NF-κB activation by promoting NEMO SUMOylation^29^.

Our study provides a strong case that amphioxus is a useful model for investigating the complex biology of vertebrate *PIAS* genes. Since this study only focuses on the NF-κB pathway and has not yet investigate SUMO E3 ligase activity, there will be more discoveries from further comparative analyses with the amphioxus as the model.

## Materials and Methods

### Animals, embryos and cells

Adult Chinese amphioxus (*Branchiostoma japonicum*) were obtained from Qingdao, China, and reared in aquaria with circulating filtered sea water. Embryos of different stages (zygote, 16 cells, blastula, gastrula, neurula and larva) were collected during the breeding season. HEK293T and HeLa cells were grown in Dulbecco’s modified Eagle’s medium supplemented with 10% fetal calf serum.

### Cloning of amphioxus PIAS cDNAs

PIAS orthologs were identified from the *B. floridae* and *B. belcheri* genomes. Based on the sequences, Gene-specific primers were designed to clone ORF sequences of bPIASv1 and v2 from cDNA libraries of the *Branchiostoma japonicum*. Subsequently, full-length cDNA sequence of bPIASv1 was obtained by performing 5′-RACE-PCR and 3′-RACE-PCR according to the manufacturer’s protocol using the Gene RACE Kit (Invitrogen).

### Expression profiles detected by quantitative real-time PCR

qRT-PCR was performed to determine the mRNA expression profiles of two bPIAS variants. For immune challenge analyses, bacterial cell wall component LPS (4 mg/ml, from *E. coli* B4; Sigma-Aldrich) or LTA (4 mg/ml, from *S. aureus*; Sigma-Aldrich) was injected into the intestines of adult amphioxus (20 μl per animal), the unchallenged animals were used as control. Intestines from five individuals were collected at 0, 1, 2, 4, 8, 12 and 24 h post-injection as a single sample. For tissue distribution analyses, tissues of muscle, skin, gill, ovary, hepatic caecum, intestine and endostyle were harvested from healthy adult amphioxus. For embryonic developmental stages analyses, Embryos of the associated stages (zygote, 16 cells, blastula, gastrula, neurula and larva) were collected and frozen in liquid nitrogen until use. Total RNA of infected samples, multiple tissues and developmental stages of amphioxus was extracted using TRIzol (Invitrogen) and digested with DNaseI (Promega), then subjected to reverse transcription (TOYOBO). qRT-PCR was conducted using SYBR PrimeScript qRT-PCR kit (Takara) and LightCycler 480 (Roche). All samples were performed under the following conditions, 1 min at 95 °C followed by 40 cycles of 15 s at 95 °C, 20 s at 60 °C, and 20 s at 72 °C. Data was quantified using the 2^−ΔCt^ method based on C_t_ values of bPIASv1, v2 and GAPDH from two parallel experiments done in triplicate. Primers used for qRT-PCR are listed as follows,

bjPIASv1-F: AATGTTACTCCAGTCCATCTCT

bjPIASv1-R: ACCACCGACAACTGCTTA

bjPIASv2-F: ATTCCCGACAGCCATACG

bjPIASv2-R: GCAGCAGAGACAGTAGGT

bjGAPDH-F: TTCACCACCATCGCCAAG

bjGAPDH-R: CTTCTCATACTTCTCCTCGTTCAC

### Plasmid construction

For the expression of bPIASv1, v2 and their truncated mutants in HEK293T cells, PCR fragments encoding amino acids 1–600, 1–115, 116–315, 1–315, 316–600, 1–382, 383–600, 471–600 of bPIASv1, 1–547, 316–547, 383–547, 471–547 of bPIASv2, were fused with Flag tag, and inserted into the expression plasmid pcDNA3.1(+) (Invitrogen) or directly inserted into pCMV-Myc vector (Clontech). For the study of subcellular localization, full-length bPIASv1, v2 and the indicated fragments described above were fused with EGFP, and inserted into the expression plasmid pcDNA3.1(+). The full-length of bIκB (1–350 aa) and bMyD88 (1–296 aa) were fused with HA tag, and subcloned into the expression plasmid pcDNA3.0 (Invitrogen). PCR fragments encoding amino acids 1–755, 1–348, 349–457, 458–755, and 349–755 of bRel were also fused with HA tag, subcloned into the expression plasmid pcDNA3.0 and designated as bRel-WT, bRel-(N + RHD), bRel-(IPT), bRel-(C), bRel-(IPT + C). The full-length of bRel was fused with the yeast Gal4 DNA binding domain (DBD), subcloned into the expression plasmid pCMV-BD (Clontech) and designated as Gal4-bRel. The full-length of bTICAM-like (1–864 aa) was inserted into pCMV-Myc vector. Vectors containing full-length human MyD88 and human TRIF fused with Flag tag were provided by Dr Tang’s laboratory (Institute of Biophysics, Chinese Academy of Science). Vectors containing full-length human PIAS family members fused with Myc tag were provided by Dr li’s laboratory (School of Life Sciences, Sun Yat-sen University). Vector containing full-length human p65 fused with Flag tag was preserved in our lab. The site-directed mutants were constructed according to the QuikChange^®^ Multi Site-Directed Mutagenesis Kit (Stratagene) and verified by DNA sequence analysis.

### Immunofluorescence imaging

Hela cells were planked with lower cell density on coverslips (10 mm × 10 mm) in a 24-well plate. After 16–24 h, cells were transfected with 500 ng indicated expression plasmids by jetPEI (PolyPlus-transfection) according to the manufacturer’s instructions. At 24 h post transfection, cells were washed twice in PBS, fixed in 4% formaldehyde solution for 15 min, and permeabilized by washing three times in PBST (0.05% Tween-20 in PBS). After blocking with 5% BSA in PBST at room temperature for 1 h, cells were further incubated with 1 μg/ml mAb for 1 h, washed three times in PBST, and incubated with the second antibody for 1 h. Following triple washing in PBS, cells were labeled with 0.2 μg/ml DAPI for 5 min, washed three times in PBS, mounted in MOWIOL R4–88 Reagent (Calbiochem) and photographed with a ZESSI Axio vision 4 microscope.

### Luciferase reporter assay

HEK293T cells were seeded in 48-well plates. After 16–24 h, cells were transfected with 500 ng/well mixed expression plasmids, which consist of the indicated amount of expression vectors, 5 ng/well of Renilla luciferase reporter plasmid pRL-TK (Promega) to allow normalization of data for transfection efficiency, and 50 ng/well of the NF-κB response promoter luciferase reporter plasmid pNF-κB-Luc (Stratagene) or pFR-Luc luciferase reporter (Clontech), which contains 5 × yeast Gal4 DBD binding elements, and the corresponding empty vectors. For cytokine stimulation, 50 ng/ml TNFα human recombinants (Sigma-Aldrich) was added to the cell medium at 24 h post transfection as described above, and cells were incubated for an additional 7 h. All Samples were measured by dual-luciferase^®^ reporter assay system (Promega).

### Coimmunoprecipitation assay

HEK293T cells seeded in six-well plates were transfected with 4–6 μg expression plasmids. At 20–36 h post-transfection, whole-cell extracts were prepared by using Co-IP lysis buffer (50 mM Tris, pH 7.4, 150 mM NaCl, 1% Nonidet P-40, 0.5% deoxycholic acid sodium salt and cocktail protease inhibitor (Roche)) and incubated with primary antibodies at 4 °C overnight, then incubated with Protein G Sepharose (Roche) at 4 °C for 4–6 h. Following triple washing in lysis buffer, analysis was conducted using SDS-PAGE followed by western blot with enhanced chemiluminescence reagent (Amersham Pharmacia, Finland). Monoclonal antibodys against HA (1:5000), FLAG (1:1000), Myc (1:7000) epitope tag and the goat anti-mouse secondary antibody (1:5000) were purchased from Sigma. Mouse monoclonal antibody against human NF-κB p65 was purchased from Cell Signaling Technology.

### Data deposition

Sequences of bPIASv1 and v2 have been deposited in the GenBank database under accession numbers KY249202 (bPIASv1) and KY249203 (bPIASv2).

## Electronic supplementary material

Phylogenetic analysis of PIAS family members. Related to Figure 1.
