# Supplementary material for: The conserved ancient role of chordate PIAS as a multilevel repressor of the NF-κB pathway
Source: Sci Rep. 2017 Dec 6;7:17063. doi: 10.1038/s41598-017-16624-7 (PMC5719053; doi:10.1038/s41598-017-16624-7)
Supplement: Supplementary file 1 — Phylogenetic analysis of PIAS family members. Related to Figure 1. [file 41598_2017_16624_MOESM1_ESM.pdf]

The conserved ancient role of chordate PIAS as a multilevel repressor of the NF-κB pathway

Ruihua Wang, Shengfeng Huang, Xianan Fu, Guangrui Huang, Xinyu Yan, Yingqiu Li and Anlong Xu

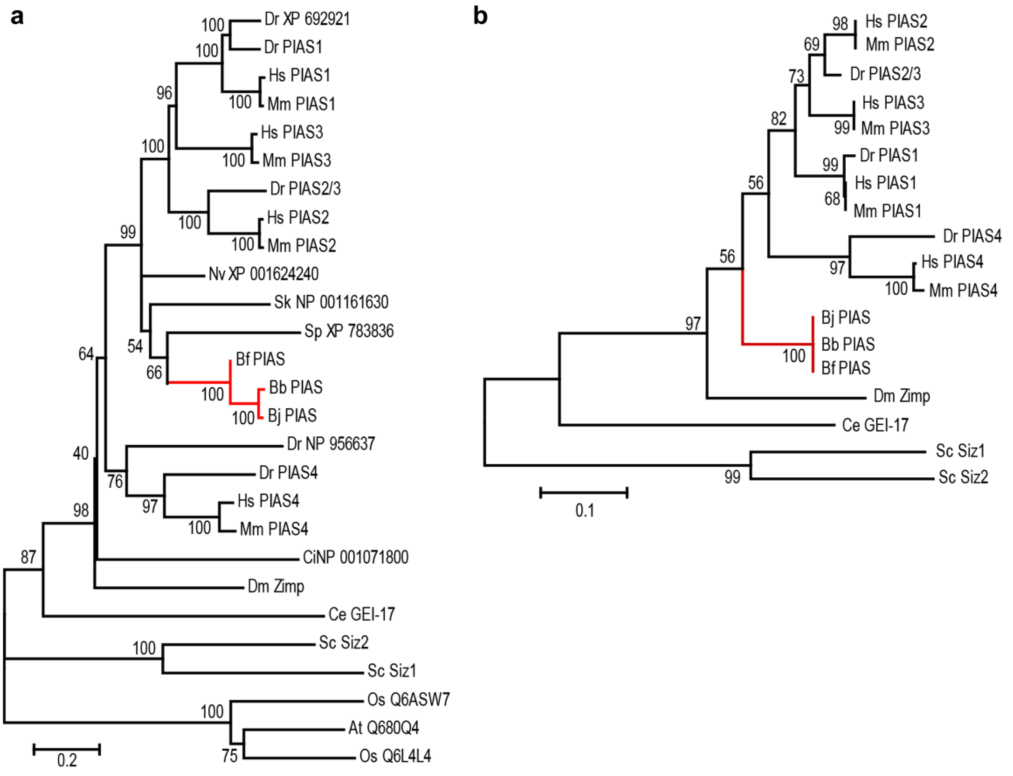

Supplementary figure 1. Phylogenetic analysis of PIAS family members. Related to Figure 1. (**a**) A phylogenetic analysis based on the full length of PIAS proteins. Because of the different rates of evolution in the non-conserved regions, the lineage of vertebrate PIAS4 was pulled away from vertebrate PIAS1, 2 and 3. If we used the most conserved domain in PIAS proteins, the RLD domain, for phylogenetic analysis, we can recover the correct tree topology shown in (**b**), in agreement with "one orthologue in basal chordates, and four paralogs by 2R-WGD". Note that the synteny analysis confirms this conclusion.
